# Review of cancer cell volatile organic compounds: their metabolism and evolution

**DOI:** 10.3389/fmolb.2024.1499104

**Published:** 2025-01-07

**Authors:** Takeshi Furuhashi, Kanako Toda, Wolfram Weckwerth

**Affiliations:** ^1^ NUS Environmental Research Institute, National University of Singapore, Singapore, Singapore; ^2^ Department of Oral Health Sciences, Health Sciences, Saitama Prefectural University, Koshigaya-shi, Japan; ^3^ Vienna Metabolomics Center (VIME), University of Vienna, Vienna, Austria; ^4^ Molecular Systems Biology (MOSYS), Department of Functional and Evolutionary Ecology, University of Vienna, Vienna, Austria; ^5^ Health in Society Research Network, University of Vienna, Vienna, Austria

**Keywords:** cancer evolution, cellular metabolism, lipid peroxidation, multifunctional enzyme, ROS, VOCs (volatile organic compounds)

## Abstract

Cancer is ranked as the top cause of premature mortality. Volatile organic compounds (VOCs) are produced from catalytic peroxidation by reactive oxygen species (ROS) and have become a highly attractive non-invasive cancer screening approach. For future clinical applications, however, the correlation between cancer hallmarks and cancer-specific VOCs requires further study. This review discusses and compares cellular metabolism, signal transduction as well as mitochondrial metabolite translocation in view of cancer evolution and the basic biology of VOCs production. Certain cancerous characteristics as well as the origin of the ROS removal system date back to procaryotes and early eukaryotes and share commonalities with non-cancerous proliferative cells. This calls for future studies on metabolic cross talks and regulation of the VOCs production pathway.

## 1 Introduction

A potential relationship between odors emitted by the human body and malignant diseases (e.g., cancer) is an intriguing current topic that has been investigated since Hippocrates’ time (Adam, 1947). VOCs (volatile organic compounds) are the main components of such odors, and a major advantage of analyzing VOCs in clinics is that it represents a non-invasive approach. The odor of various specimens (breath, urine, feces, the body surface, sweat) has been used as a sample source, and various types of VOCs (e.g., hydrocarbons including alkanes and alkenes, aldehydes, alcohols, ketones, aromatics, carboxylic acids, esters, ethers, nitrogen- or sulfide-containing volatile compounds) have been reported (Filipiak et al., 2016; Gouzerh et al., 2022). In particular, VOCs application for cancer detection is an emergent topic because cancer has one of the highest mortality rates and most severe patient suffering. At the same time, cancer care costs have exploded (Mariotto et al., 2020).

Further advancing the application of VOCs in cancer detection and care requires developing both basic biology and applied science approaches. To date, many of the analyses have solely involved VOCs profiling. Little is known about the enzymes related to VOCs production, VOCs metabolism control, environmental cues influencing VOCs metabolism, and the gene expression involved in VOCs metabolism. Moreover, the essential reasons for cancer cells to emit cancer-specific VOCs as well as the evolutionary origin of VOCs metabolism have never been fully addressed. Here we review cancer-dependent VOCs metabolism and evolution to summarize and improve our basic understanding. This approach will eventually contribute to non-invasive VOCs analysis, enabling the detection of cancer-specific biomarkers and pointing to direct therapeutic strategies.

## 2 Basic biology of VOCs

### 2.1 Cancer evolution and cellular VOCs production

Studying the production of cancer VOCs benefits from reviewing cancer evolution itself. Cancer is clearly observed only in multicellular organisms (animals, plants, fungi), mostly in the animal kingdom (Aktipis et al., 2015). Cellular differentiation is not stable in fungi, and abnormal growth in fruit bodies has been detected (e.g., Ascomycota and Basidiomycota). Dedifferentiation of hyphal cells and subsequent fungal fruit renewal are characteristic features (Umar and Van Griensven, 1999), but metastasis has not yet been observed and no vascularization occurs. In plants, cacti fasciation or cristation and crown galls can be formed. These are often provoked by bacterial infection with the upregulation of hypoxia responsive genes (Kerpen et al., 2019) and sometimes even by parasitization by parasitic plants correlating with upregulation of auxin and cytokinin (Furuhashi et al., 2014). Similar to animal cancer, vascularization has also been observed in plant gall tissues. Only “metastasis-like” phenomena have been reported (Braun, 1943), but metastasis as documented in animals has not been documented in plants. Plant cells are less mobile/flexible due to the presence of more rigid cell walls, and a circulation system such as blood vessels in animals is missing. In the animal kingdom, cnidarians were probably the first phylum in which neural tissue, muscular tissue and mesoderm appeared in evolution (Aktipis et al., 2015).

ROS are divided into two categories encompassing radical and non-radical forms (Chiurchiù and Maccarrone, 2011). ROS include not only the widely known superoxide radical, hydrogen peroxide, and the hydroxyl anion, but also peroxinitrite and the sulfonyl radical, which contain nitrogen (RNS: reactive nitrogen species) and sulfide (RSS: reactive sulfide species), respectively (Moulian et al., 2001; Giles and Claus Jacob, 2002). ROS signaling is major cellular trend. For example, physiological signaling functions of ROS are reported in tissue, iPSCs regeneration, and pluripotency of stem cell (Sinenko et al., 2021). In fact, a change in cellular ROS level is associated with a metabolic shift between glycolysis and mitochondrial respiration during cellular differentiation process.

ROS function in cellular signal transduction, called redox signaling, is currently an emergent topic. Mostly, ROS influence or inactivate proteins, such as enzymes and transcriptional factors, but there are cases that ROS is required for cellular function, such as disulfide bond formation. Examples are redox regulation of the insulin signaling pathway (e.g., PTEN inactivation), transcription factors (e.g., NRF2), epigenetics (e.g., DNA methylation), mitochondrial energy metabolism (e.g., inactivation of aconitase), circadian rhyme (e.g., sleep-wake rhythm) and proteostasis (e.g., disulfide formation) (Lennicke and Cochemé, 2021).

In the case of plant metabolism, not only ROS but also RNS is involved in cell signaling and plant-microbe interaction, in view of resistance against pathogen/symbiont and program cell death (PCD) (Khan et al., 2023).

**FIGURE 1:**
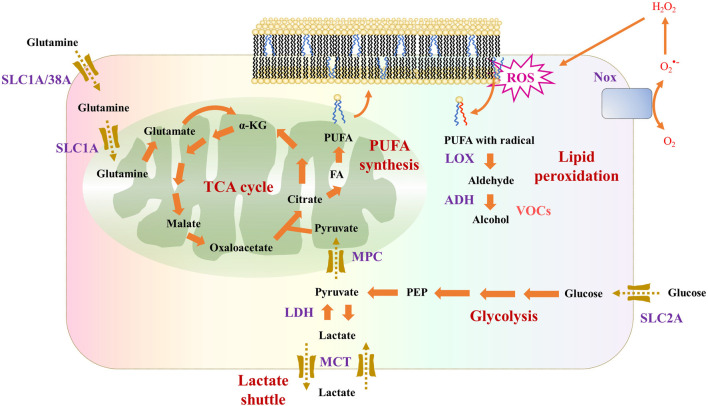
Cellular metabolic pathways potentially related to VOCs production. Glucose and glutamine are two main energy sources for cancer cells. ROS generated by Nox (NADPH oxidase), for example, will cause lipid peroxidation and eventually generate VOCs such as volatile alcohols.

In VOCs production in cells, ROS (reactive oxygen species) play a role in catalytic peroxidation and are key for our understanding of this process (Figure 1). H_2_O_2_ exposure leads to oxidative stress and eventually to VOCs production in A549 lung cancer cell lines *in vitro*, making ROS a conspicuous source of VOCs production (Fenn et al., 2022).

The consequences, and the ROS-mediated signal transduction, differ depending on ROS concentration as well as its source: low ROS tend to promote cancer proliferation (e.g., PI3K/Akt activation through PTEN inhibition) and invasion (e.g., NF-κB activation by PP2A inhibition), whereas high ROS lead to oxidative stress, apoptosis (p38 activation through ASK-1) as well as metastasis inhibition (Foo et al., 2021). Moreover, ROS induce not only lipid peroxidation but also DNA damage, autophagy and ferroptosis (Sainz et al., 2012; Su et al., 2019).

ROS can be produced in several sites inside the cell and can be derived from both endogenous and exogenous sources (e.g., pathogens and radiation) (Dayem et al., 2010). As an endogenous source, in humans, O_2_^•-^ (ROS) are generated not only by incomplete reaction in the mitochondrial electron transport system (ETC), but also by NADPH oxidase on plasma membranes, cytosolic xanthine oxidase, peroxidase and cytochrome P450 (Bechtel and Bauer, 2009; Collin, 2019). As there are several ROS production sites, ROS has been considered to be randomly diffused, but the possibility exists that cells can strictly control ROS source activation, localization as well as the amount and duration of ROS production (Herb et al., 2021).

Generated ROS can provoke oxidative stress, usually reflecting an imbalance between antioxidant systems and various pro-oxidants, which can be related to disease. Accordingly, loss of this balance leads to oxidation of biomolecules. In general, 90% of the oxygen uptake by cells is used for energy production by mitochondrial respiration, while the remaining 10% is consumed for non-enzymatic chemical reactions (Calenic et al., 2015). Considering that peroxidation constitutes a mixture of non-enzymatic and enzymatic chemical reactions, the role of ROS in the peroxidation pathway is not negligible (Higdon et al., 2012).

Regarding oxidation targets, proteins, nucleic acids and lipids can be targets for ROS. As an example, Arg and Pro in proteins can be converted into γ-glutamyl semialdehyde by protein oxidation by H_2_O_2_, indicating that lipids as well as proteins and nucleic acids could be targets for ROS-mediated oxidation (Calenic et al., 2015). Another piece of evidence is that some cancer cell VOCs contain nitrogen and sulfide, e.g., pyrrole and dimethyl sulfide (Filipiak et al., 2010; Mochalski et al., 2018). The data suggest that some VOCs are probably derived from amino acid oxidation because sulfide is present only in proteins, not in nucleic acids or fatty acids. Consistent with this, a recent study showed that methanethiol is converted into dimethyl sulfide in cancer due to blockage of SELENBP1 (Selenium-binding protein 1) by mutation (Philipp et al., 2023). Presumably, methionine is an origin of methanethiol (Yeretzian et al., 2017).

In the case of DNA bases, such bases are oxidized by radicals, leading to deamination (Cooke et al., 2003), or aldehydes produced by lipid peroxidation react with DNA (Medeiros, 2019). Lipid peroxidation typically involves oxidation of fatty acids in lipid membranes, especially PUFA (polyunsaturated fatty acids). It can generate aldehydes, which are toxic reactive compounds for cells, so that aldehydes need be converted into more stable forms such as smaller-sized volatile compounds (e.g., alcohol and carboxylic acids) (Ayala et al., 2014).

From an evolutionary viewpoint, several aspects of VOCs production merit discussion. These include ROS removal systems together with lipid peroxidation, the metabolisms that potentially regulate VOCs production (e.g., energy metabolism), metabolic interactions between cells, and responses to hypoxia.

As an ROS removal system, SOD (superoxide dismutase) is one of the most important enzymes for antioxidant defense, and SOD-based antioxidative systems already evolved in single-cell organisms (e.g., bacteria and protozoans). Here, the ocean evolution event involving an euxinic ocean [e.g., Great Oxygenation Event (GOE) and Neoproterozoic Oxygenation Event (NOE)] appears to be associated with the evolution of sulfur and oxygen metabolisms (Feelisch et al., 2022). Interestingly, one phylogenetic study on cyanobacteria antioxidant enzymes showed that the type of metal (e.g., Cu and Zn) incorporated into SOD metalloenzymes differed based on the distribution and availability in the environment at each paleontological stage (Boden et al., 2021). CuZnSOD was probably already acquired by non-marine cyanobacteria in the Archaean, which was prior to the GOE. In contrast, in NOE, the utilization of FeSOD and MnSOD increased concomitantly with the requirement for an ROS defensive system. Antioxidative systems are present in fungi (Aguirre et al., 2006). In the unicellular fungus *Schizosaccharomyces pombe*, this system involves a prokaryotic-type multistep phosphorelay coupled to a stress-response MAP kinase pathway and an AP-1 type transcription factor. Many phosphorelay sensor kinases, antioxidant enzymes and antioxidative secondary metabolites are apparently present in filamentous fungi (e.g., *Aspergillus nidulans*), which express Nox-like enzymes producing ROS. Importantly, ROS mediated AP-1, a redox regulated transcriptional factor, is well known in cancer ROS signaling (Liou and Storz, 2010). Moreover, Blackstone (2000) reported that the evolution of a redox system can be implicated with multicellularity, whereby a hypoxic effect is avoided by terminal differentiation based on ROS signal transduction. In addition, the thylakoid membrane can be a site that generates ROS during photosynthesis in lower plants and algae, using enzymes for an antioxidative defense system (e.g., superoxide dismutase, catalase, ascorbate peroxidase, glutathione reductase) (Rezayian et al., 2019). Some of these enzymes are used in higher plants as well (Huang et al., 2019).

The lipoxygenase (LOX) family related to lipid peroxidation is also observed in prokaryotes, and two animal LOX superfamilies (LOXL2/L3/L4 and LOX/L1/L5) presumably evolved during metazoan evolution (Grau-Bové et al., 2015). ROS-mediated LOX-based lipid peroxidation itself is common, but downstream of LOX the pathways differ between organisms. For example, plants use hydroperoxide lyase (HPL) belonging to the CYP 74 family, generating COX (Riley et al., 1996), whereas HPL has not been reported in animals.

Regarding peroxidases, phylogenetic studies on animal peroxidase infer the presence of at least five clades and vertebrate peroxidasins in which the metalloprotein peroxidase-cyclooxygenase superfamily (related to prostaglandin synthesis) belongs (Soudi et al., 2012).

Certain radicals can be converted into stable VOCs compounds by multiple functional enzymes. Accordingly, previous research on lung cancer cell lines A549 reveals that some cancer VOCs can be catalyzed by ADH (Furuhashi et al., 2023), which is known to catalyze various types of aldehydes into alcohols and vice versa (Boleda et al., 1993). Cancer cells critically require a set of multifunctional enzymes and/or isozymes to cope with unexpected ROS generation and its toxicity, lending plasticity to cancer cells. A link between detoxification based on enzymatic promiscuity and multifunctionality is intriguing, while the presence of multifunctionality and isozymes (e.g., ADH and ALDH families) would make it difficult to specify target enzymes and to make a knockout mouse for VOCs metabolism research. Another technical difficulty is identifying VOCs products from *in vitro* enzyme assays because the product of enzymatic reactions can be volatile. Derivatization can be applied to improve sensitivity and selectivity. Considering that *in vitro* enzymatic assays must be conducted under aqueous conditions, this involves either (i) using a derivatization reagent that can function in the presence of water (e.g., chloroformate for short chain fatty acids) (Furuhashi et al., 2018), or (ii) transferring VOCs products into an organic solvent phase prior to derivatization (e.g., *Trans* 2 hexenol to assess ADH enzymatic activity) (Furuhashi et al., 2023).

ROS-based radical compounds in cancer cells are partly generated non-enzymatically, producing a variety of radicals (e.g., different length and saturation of fatty acids). Confusingly, in some studies, purely non-enzymatic chemical reactions, spontaneous reactions on enzymes, and low specificity enzymatic reactions are recognized as non-enzymatic reactions.

Multifunctionality of enzymes, e.g., protease (López-Otín and Bond, 2008), has been previously studied under the heading of enzyme promiscuity (Piedrafita et al., 2015). This has been classified into three groups: substrate promiscuity (one enzyme can catalyze several different substrates), catalytic promiscuity (an enzyme can catalyze the same substrate into different products), and conditional promiscuity (an enzyme can catalyze different substrates and produce different products due to substrate concentration change and translational modification). As an interesting example of enzymatic multifunctionality, plant CYP450 promiscuity occurs in diterpenoid metabolism (e.g., in monocots and conifers), and also in sesquiterpenoid metabolism as a detoxification of endogenous toxins, so-called phytoalexins (e.g., capsidiol in Solanaceae) (Werck-Reichhart, 2023). Multiple functions are also observed in AhR (aryl hydrocarbon receptor), which is a xenobiotic-receptor to eliminate exogenous toxic or harmful chemical compounds (Tomkiewicz et al., 2024).

Conformational flexibility – the ability of conformation change between different substrates, observed in various organisms including prokaryotes – is a key to understand enzymatic promiscuity providing multifunctionality (Petrović et al., 2018). Conformational flexibility involves acquiring additional promiscuous catalytic activities or even completely new activities on scaffolds that were previously non-catalytic, for example, by gene duplication. Moreover, coenzymes (i.e., NADH and NADPH) are required for conformational change (Plapp, 2010), which could be influenced by the environment (e.g., balance between oxidation and reduction).

There is an interesting case that ROS can also induce multifunctionality of enzymes, as seen in example of peroxiredoxins (Detienne et al., 2018). ROS can alter cysteine residues into cysteine sulfinic acid. A peroxidase activity is inactivated and then it turned into a multimeric complex with chaperone function. While, still there is no evidence that cellular VOC production is influenced by ROS-induced enzymatic function change.

Finally, the regulation of these enzymes differs under various environmental conditions and can expand multifunctionality and also provide further plasticity to cellular metabolism. For example, ADH and LDH can be upregulated under hypoxia in plants (Lin et al., 2017), indicating that searching for hallmarks related to cancer VOCs production is crucial.

### 2.2 Cellular metabolism and VOCs production

Considering ROS as a result of a metabolic shift between glycolysis and mitochondrial respiration, energy metabolism has been of particular interest. Rapid cell proliferation is a key cancer characteristic that affects metabolism. This *per se* involves how to utilize metabolites (e.g., glucose) as an energy source under certain environmental conditions. Regarding metabolism evolution, two different metabolic effects already appeared in single-cell bacteria and protozoans (e.g., *Saccharomyces*). One is the Pasteur effect in which yeast glycolysis is suppressed and the TCA cycle is extensively used under aerobic conditions. This was reported by Pasteur in 1861, originally called the Pasture reaction by Warburg in 1926 (Racker, 1974). This effect was later commonly observed in human tissue as well (Ramaiah, 1974).

Secondly, when cells are under nutrient-rich conditions (i.e., high glucose), the glycolysis pathway needs to be activated. At the same time, an oxygen supply is also required for the subsequent TCA cycle and oxidative phosphorylation. Respiration ability and glucose availability are related to each other (Dashko et al., 2014). For instance, if the glucose supply is in excess or the oxygen supply is relatively insufficient, cells suppress oxygen consumption in mitochondria and the end product of glycolysis turns toward the fermentation process. Consequently, cells tend to produce energy without using oxygen and utilize NADH generated in glycolysis for the fermentation process. Today, this phenomenon (particular in yeast) is recognized as the Crabtree effect (Malina et al., 2021). Nonetheless, Crabtree originally followed the Warburg study (i.e., aerobic glycolysis) by using a rat carcinoma and calculating excess fermentation from respiration Q_O2_ and anaerobic glycolysis Q_M_ (Q_M_ -2x Q_O2_) (Crabtree, 1929), stating that glycolytic activity exerts a checking effect on respiration. Although Crabtree used cancer tissue and used the term “fermentation” (i.e., lactate was recognized as a waste product), the term “Crabtree effect” nowadays pertains more to the alcoholic fermentation process in yeast (i.e., ethanol production) than to lactate metabolism in metazoans (exclusively cancer cells). Historically, the term Crabtree effect was applied to avoid confusion with the Pasteur effect. The former has, in turn, been used rather for yeast fermentation metabolism than for cancer. De Deken (1966) suggested using the term Crabtree effect instead of “contre-effet (counter effect in French) Pasteur” to explain yeast fermentation under high glucose conditions. Regarding the Warburg effect, glycolysis is active both under aerobic and anaerobic conditions. Accordingly, oxygen concentration changes do not suppress glycolysis (Warburg et al., 1927). An evolutionary origin of the Warburg effect is still enigmatic, and the TCA cycle and oxidative phosphorylation are not suppressed even under hypoxia in cancer, which is far different from single cell metabolism. Among these three metabolic effects, the Warburg effect appears to be closely related to cancer proliferation. Note, however, that the Warburg effect can also be observed in proliferating animal cells including non-cancerous cells (e.g., immune T-cells) (Abdel-Haleem et al., 2017).

In a previous study comparing non-cancerous proliferating lung cells (HLB) and cancerous A549 lung cells, both cell types showed extensive glucose use under aerobic conditions, but the VOCs profiles differed (Furuhashi et al., 2020). That insight suggested that cancer-specific VOCs production is not caused solely by the Warburg effect. At the same time, a positive influence of lactate on cancer VOCs production was investigated by comparing lung primary cells, non-cancerous proliferating cells and cancer cells (Furuhashi et al., 2023). Lactate is recognized not only as an energy metabolite but also as an extracellular messenger by interacting with the GPR receptor, e.g., GPR81 (Brown and Ganapathy, 2020). This makes the influence of lactate signaling on VOCs production an interesting future research topic.

Extensive utilization of glycolysis leads to production of pyruvate, and subsequently to lactate by LDH. This is characteristic in higher animals (typically cancer) but has also been observed in prokaryotes, e.g., *Lactobacillus* metabolism. The conversion of pyruvate, however, differs among organisms. Some bacteria and protostomes can produce opines, which are a conjugation of amino acids with pyruvate by dehydrogenases (e.g., opine dehydrogenase) (Sato et al., 1993). Kerpen et al. (2019) recently stated that plant crown gall contains such opines that appear to be related to plant cell proliferation. This phenomenon is thought to help avoid the accumulation of individual metabolites.

Lactate as an end-product is reusable and can be translocated. Cells extensively utilize glucose and produce lactate in glycolytic status, while neighboring oxidative status cells uptake lactate and convert it into pyruvate by LDH-B to generate ATP via the TCA cycle. This has been called the lactate shuttle (Brooks, 2018). Regarding lactate utilization, recent myotube flux analysis indicates that the carbon of lactate can be ultimately used as a substrate for glycogen as well as lipids (Lund et al., 2018).

Recent spatial transcriptomics revealed different cells in the TME (tumor micro environment), consisting of heterogeneous cancer cells including cancer stem cells and non-cancer cells (e.g., CAF, cancer-associated fibroblasts) (Sahai et al., 2020). In TME, the lactate translocation from CAF to cancer cells (Li et al., 2021) is probably important for cancer cells. Interestingly, this lactate translocation can provoke metabolic reprogramming (Li et al., 2021). Such a translocation also occurs between glia cells and neurons, e.g., primary cells (Liu et al., 2017). Further investigation of metabolite translocation and the relationship with VOCs would benefit from flux analysis using isotope labels as well as modeling and simulation (e.g., ENGRO model and FBA analysis) (Damiani et al., 2017). Nevertheless, few studies have been conducted on VOCs flux analysis, possibly because of the difficulty to detect VOCs generated by lipid peroxidation.

From an evolutionary perspective, metabolite translocation between cells appeared in prokaryotes and single-cell eukaryotes, as evident in microbial interactions in food fermentation (e.g., yogurt, Korean kimuchi, Japanese miso, and the Thai food nham) (Sharma et al., 2020). Such translocation also occurss in metabolic interactions involving host-pathogen interactions in the gut (Llibre et al., 2021). Metabolic interactions might be correlated with the acquisition of an acid tolerance system (e.g., acetic acid tolerance) (Lynch et al., 2019). Mutually beneficial metabolic interactions are well known, for example, interactions between lactic acid bacteria and yeast in maize fermentation (Chaves-López et al., 2020). Note that competitive relationships are also possible, such as in the relationship between aerobic *Acetobacter pasteurianus* and anaerobic *Lactobacillus helveticus* in Chinese vinegar (Xia et al., 2022). Utilization of lactate produced by anaerobic bacteria (uptake), then converted into pyruvate and incorporated into the TCA cycle by aerobic bacteria, is reminiscent of the lactate shuttle between cancer cells.

Glutamine is another key metabolite in cancer cell metabolism. It generates energy from amino acids instead of sugars and is termed glutaminolysis (Yang et al., 2017). Glutamine is the most abundant amino acid in blood and muscle, and can be an alternative energy source instead of sugars (Yang et al., 2017). Furthermore, in cancer cell line studies, glutamine deprivation reduced VOCs production in cancer cells. That production level was not recovered by adding sugars or lactate. Active glutaminolysis can be observed in non-cancerous proliferative cells as well, for example, in neural progenitor cells (NPCs) for neocortex development and basal progenitor expansion (Gkini and Namba, 2023). Another example is that pluripotent stem cells (hPSCs) use glutamine instead of pyruvate as a main energy source, whereas cardiomyocytes can utilize lactate to synthesize pyruvate and glutamate (Tohyama et al., 2016).

Focusing solely on single metabolic characteristics (e.g., Warburg effect) does not shed light on cancer-specific metabolisms. Accordingly, the next step must involve regulating the metabolic status in certain environments. For example, there is a crossroad between glycolysis and glutaminolysis known as the CtBP-Sirt4-GDH axis (Tomaselli et al., 2020; Li et al., 2022). In particular under high glucose conditions, CtBP (C-terminal binding protein) dimerizes and binds to the Sirt4 (Sirtuins4) promoter and represses its expression; this consequently increases glutamate dehydrogenase (GDH) activity (Wang et al., 2018a; Bai et al., 2021). Regarding the relationship between lactate and glutaminolysis, lactate converted into pyruvate inhibits the hypoxia-inducible factor prolyl hydroxylase (PHD) and HIF-2a stabilization and c-Myc transactivation. This in turn induces upregulation of the glutamine transporter ASCT2 and of glutaminase GLS1, which is the first step of glutaminolysis in mitochondria (Pérez-Escuredo et al., 2016). Nonetheless, few investigations have been conducted and an influence of these crossroads on VOCs production might be promising future work.

Responding to hypoxia is another hallmark of cancer: metastasis (e.g., motility), invasion and vascularization can be induced by hypoxia (Wang et al., 2007; Balamurugan, 2016). Signal transduction for responding to hypoxia is by HIF, and HIF can promote FFA synthesis. HIF1α affects glycolysis and lactate transport, while HIF2α influences the glutamine transporter (i.e., glutaminolysis) and fatty acid synthesis (Yoo et al., 2020). Moreover, the L-2-hydroxyglutarate (L-2-HG) produced by lactate dehydrogenase A (LDHA) and malate dehydrogenase (MDH) under hypoxia contributes to regulating histone and DNA methylation levels. This is because L-2-HG inhibits those epigenetic modification enzymes that require glutamine-derived α-ketoacid as a cofactor. This insight suggested a potential role of glutaminolysis in cancer epigenesis, one that might promote cancer plasticity.

**FIGURE 2:**
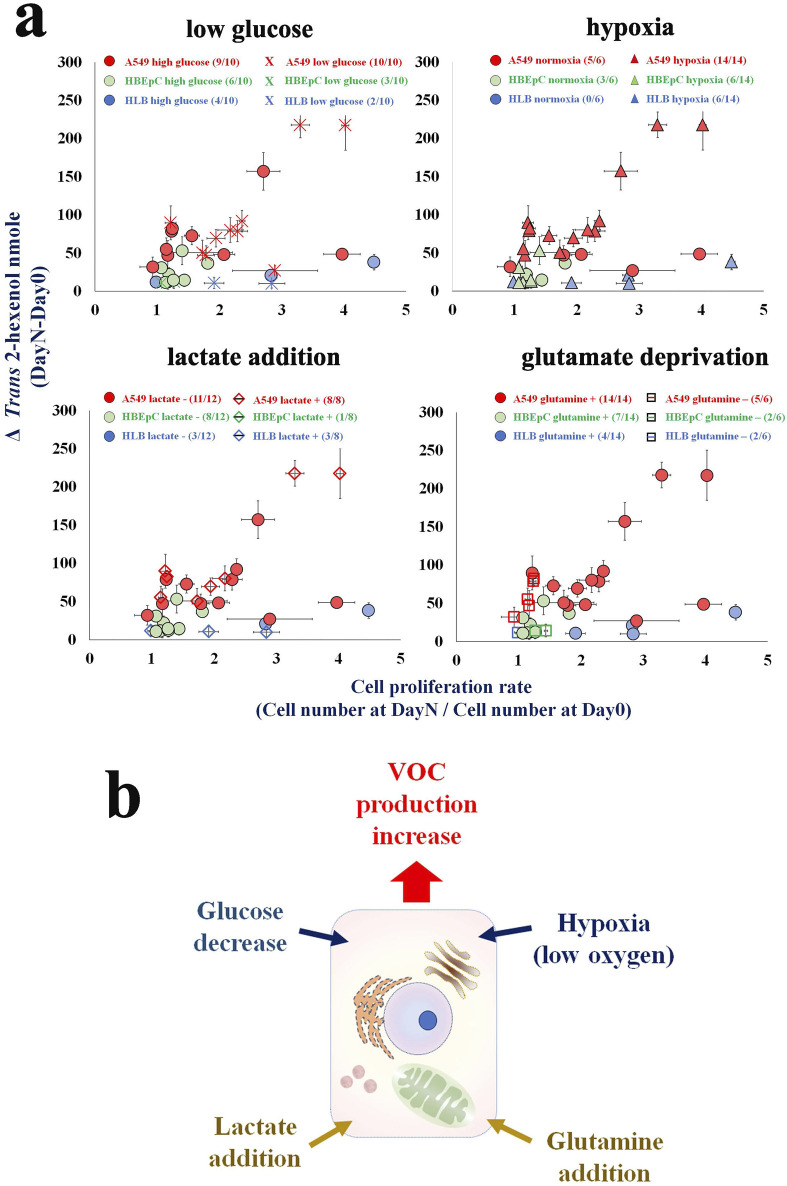
**(A)** An influence of condition to cellular VOC production (i.e., low glucose, hypoxia, lactate addition, glutamate deprivation). X axis is cell proliferation rate [Cell number (Day X/Day 0)] and Y axis is ΔVOC [trans-2-hexenol nmole (DayX-Day 0)]. Day X is either Day 2 or Day 3 [Modified from Furuhashi et al. (2023)]. **(B)** Hypothetical VOC production scheme in cancer cell. Culture cells with low glucose, hypoxia, lactate addition, glutamate addition are factors to influence VOC production.

Compared with primary cells, cancer and non-cancerous cells can proliferate under hypoxia. In addition, hypoxia can promote cancer cell VOCs production, although the underlying mechanism remains uncertain (Furuhashi et al., 2023) (Figure 2). The assumption is that ROS formation decreases under hypoxia. Note, however, that one study also showed that ROS generation was paradoxically increased with a drop in oxygen concentration, i.e., hypoxia (Zorov et al., 2014).

ROS appears to be generated in mitochondria, but the phenomenon is lost when mitochondria are isolated. This points to an extramitochondrial factor. Regarding ATP production in mitochondria, a high mitochondrial membrane potential (ΔΨ_m_ above 150 mV) increases ATP production but also increases ROS formation (Lee et al., 2001). Considering that cancer cells do not decrease energy production under hypoxia, mitochondria in cancer cells need to balance between ATP production and ROS formation, which may involve metabolic alterations in cancer cells. Finally, hypoxia can also induce ROS by Nox, subsequently activating HIF signaling, upregulating the glycolysis pathway and lactate production (typically HIF1α) (Forrester et al., 2018). It would be intriguing to determine whether the hypoxia inducible factor (HIF1) could maintain stemness of MSC by constraining metabolic reprogramming, for example, by activating anaerobic glycolysis and suppressing mitochondria respiration (Mohammadalipour et al., 2020).

### 2.3 Mitochondria and VOCs production

Mitochondria are the main organelle responding to hypoxia and possess two membranes. The outer membrane is a signaling platform where the phosphorylation of many proteins occurs, while the inner membrane is an ATP factory generating energy by TCA and ETC complexes (Grasso et al., 2020). Furthermore, the LDH and ADH complexes apparently localize onto the outer membrane in mouse liver mitochondria (Zimin and Solovyova, 2009), suggesting that mitochondria are potential VOCs production sites. In this section, we review mitochondria evolution, the response to hypoxia, as well as the organic acid (i.e., lactate) including amino acid (i.e., glutamine) transfer between cells.

Lynn Margulis in 1970 proposed that mitochondria were originally symbionts in host cells, and they are now known to have originated from the bacterial phylum α-Proteobacteria (Alphaproteobacteria) (Gray, 2012).

Consistent with this idea, α-proteobacteria (e.g., *Paracoccus denitrificans*) possess a respiratory chain similar to ETC and release superoxide as a byproduct; ROS interact with cysteine residues within transcription factors that activate prokaryotic genes (Chandel, 2015). A recent study showed that these ancestral bacteria are present in the marine environment and feature aerobic traits (Geiger et al., 2023).

Mitochondria can be classified into 5 types based on function: (i) aerobic, (ii) anaerobic, (iii) H_2_-producing mitochondria, (iv) hydrosomes and (v) mitosomes that do not generate ATP (Müller et al., 2012), although there is some room to discuss a functional continuum between them (Roger et al., 2017).

Eukaryotes diversified in the Proterozoic era, when the oxygen concentration was low, (Zimorski et al., 2019), so that the evolution of mitochondrial morphology may well be associated with the evolution of anaerobic energy metabolism in eukaryotes, e.g., adaptation to a hypoxic or anoxic environment. Eukaryotes with facultatively anaerobic mitochondria would be capable of utilizing fumarate reductase and RQ.

Recently, biochemical and genetic investigations of the α-proteobacterium *Rhodospirillum rubrum* demonstrated that UQ is a precursor to RQ (Brajcich et al., 2010). Moreover, the function of a putative methyltransferase (RquA) in the mitochondrion-related organelles of the anaerobic protist *Pygsuia* was correlated with the presence of RQ (Stairs et al., 2018).

There are several morphological types in mitochondria, including fragmented (fission), tubular and filamentous (balanced), and hyperfused (fusion) (Wang et al., 2022). Cancer tissue is heterogeneous, i.e., a mixture of cancer cells with fragmented mitochondria and cancer stem cells (CSC) with tubular mitochondria (possibly due to a high mitochondria turnover rate) (Kim and Cheong, 2020). Nothing is known about whether these mitochondrial morphological differences influence VOCs, and getting the whole picture of the VOCs production mechanism from cancer cell tissue remains a major task.

Organic acid transport between cytosol and mitochondria would be key for understanding energy metabolism (e.g., amino acid-based energy production) as well as the adaptation to hypoxia (i.e., anaerobic conditions). Typically, glutamine is initially uptaken into cytosol by SLC1A/38A on the plasma membrane and then moves into mitochondria by SLC1A (Hewton et al., 2021). In particular, a variant of SLC1A5 can be induced by HIF2α under hypoxia (Yoo et al., 2020). Glutamine is firstly converted into glutamate by GLS, then converted into α-ketoglutarate, and energy is generated by the TCA cycle. Such an amino acid-based energy production differs between organisms (e.g., glutamate, aspartate, malate, pyruvate or phosphoenolpyruvate) and could be related to the diversification of amino acid transporters in the inner mitochondrial membrane (e.g., SLC25A) to adapt to anaerobic conditions.

The SLC25 family, for example, is common in eukaryotes (e.g., *Saccharomyces*, *Caenorhabditis elegans*, *Drosophila melanogaster*, *Danio rerio*) (Byrne et al., 2023), with three characteristic contact points at the central substrate binding site (Ruprecht and Kunji, 2020). In a recent human SLC25 phylogenetic study, SLC 25 was divided into three groups based on substrates (i.e., amino acids, carboxylates, nucleotides), and SLC25 diversification could reflect intron repositioning and exon shuffling (Monné et al., 2023).

Examples include the aspartate-glutamate anti-transporter SLC25A12 (AGC) and the malate-oxoglutarate anti-transporter SLC25A11 (Amoedo et al., 2016). Between these, malate and pyruvate are emphasized in anaerobic energy metabolism. Malate is uptaken by mitochondria and utilized as an energy source in molluscs, pyruvate in algae (Chlamydomonas) (Müller et al., 2012). In cancer cells, glutaminolysis is an important energy source. Here, malate transport, typically known as the malate-aspartate shuttle (MAS), is important to maintain a high NADH/NAD ratio in mitochondria to cytosol (Borst, 2020), and AGC is potentially important to regenerate cytosolic glutathione, i.e., an antioxidant (Amoedo et al., 2016).

Pyruvate transport, in turn, involves the mitochondrial pyruvate carrier (MPC), and the expression of mpc2 and mpc3 was specified to the fermentation process (ethanol production by pyruvate decarboxylase) and to the respiration process (TCA cycle and energy production by pyruvate dehydrogenase) in yeast, respectively (Bender et al., 2015). In cancer, glutamine deprivation leads to activation of MPC, driving the TCA cycle, and can also induce the amino acid (Asp and Arg) carrier SLC1A3 and SLC37A3 expression (Jin et al., 2023). Onterestingly, MPC expression can also negatively influence cancer cell proliferation, and even MPC disruption can promote glutaminolysis (Rauckhorst and Taylor, 2016). In fact, MPC is classified into the SLC54 family, which differs from most of amino acid transport by the SLC25 family (Ferrada and Superti-Furga, 2022).

Pyruvate kinase is an enzyme in glycolysis and catalyzes phosphor enol pyruvate (PEP) into pyruvate, a process that is conserved among living organisms from prokaryotes to higher vertebrates (Oria-Hernández et al., 2006). The presence of mitochondria implicates pyruvate kinase activity in the evolutionary context. In fact, both *Entamoeba histolytica* (mitochondria-lacking parasitic amoeboid protozoan) and *Giardia intestinalis* (intestinal unicellular parasite with reduced form of mitochondria) convert phosphoenolpyruvate (PEP) to pyruvate by pyruvate:orthophosphate dikinase (PPDK) rather than by pyruvate kinase (Müller et al., 2012).

Based on enzymatic assays in the 1970s, pyruvate kinase was classified into several isoforms based on location in tissue, e.g., K, L and M, named after kidney, liver and muscle, respectively (Carbonell et al., 1973; Ibsen, 1977). Today, pyruvate kinase isoenzymes are mostly classified as type M1 and type M2, both showing an allosteric effect and being derived from alternative splicing regulated by the SMAR1 tumor suppressor (Choksi et al., 2021). PKM form homo tetramers, and the ratio between PKM1:PKM2 alters metabolism, influencing glutaminolysis and lactate production. Importantly, certain PKM also reflect tumor origin (Morita et al., 2018). Notably, hypoxia-induced HIF-1 activates PKM2 transcription, indicating that the aerobic/anaerobic transition is related to PKM isoform selection as well (Luo et al., 2011). The relationship between VOCs production and pyruvate kinase is not known and would be an intriguing topic of future research.

## 3 Conclusion

The origin of the ROS removal system dates back to prokaryotes and early eukaryotes. Cellular VOCs derived from lipid peroxidation might be linked to adapting to anaerobic conditions, and metabolite (e.g., organic acid) translocation could be a key to understanding VOCs production. A link between rapid growth and ROS generation leads to lipid peroxidation, making cancer cell VOCs an intriguing topic. Nonetheless, cancer VOC studies have left certain open questions. For example, many metabolic characteristics (e.g., the Warburg effect) that have been recognized as cancer-specific hallmarks are also occasionally common in non-cancerous proliferative cells. Future studies are required to strengthen our understanding of multifunctional enzymes (e.g., functional change induced by ROS) and the regulation of the lipid peroxidation-based VOCs production under various conditions (e.g., hypoxia).
